# Bonding Performance and Interfacial Ultra-Morphology/Nanoleakage of a Modern Self-Curing Bulk-Fill Restorative System: An *In Vitro* Study

**DOI:** 10.1055/s-0045-1804886

**Published:** 2025-03-25

**Authors:** Paula Maciel Pires, Aline Almeida Neves, Paul Farrar, Álvaro Ferrando Cascales, Avijit Banerjee, Victor Pinheiro Feitosa, Salvatore Sauro

**Affiliations:** 1 Department of Pediatric Dentistry and Orthodontics, Universidade Federal do Rio de Janeiro, Rio de Janeiro, Brazil; 2 Dental Biomaterials & Minimally Invasive Dentistry, Departamento de Odontologia, CEU Cardenal Herrera University, Valencia, Spain; 3 Research & Development, SDI Limited, Bayswater, Australia; 4 Department of Biomaterials Engineering, Faculty of Medicine, UCAM, Universidad Católica de Murcia, Campus Los Jerónimos, Murcia, Spain; 5 Conservative and Minimally Invasive Dentistry, Centre of Oral Clinical Translational Sciences, Faculty of Dentistry, Oral and Craniofacial Sciences, King's College London, London, United Kingdom; 6 Department of Operative Dentistry, University of Iowa College of Dentistry, Iowa City, Iowa, United States; 7 Department of Therapeutic Dentistry, I. M. Sechenov First Moscow State Medical University, Moscow, Russia

**Keywords:** dentin, self-curing, dental resins, microtensile bond strength, resin–dentin interface, microscopy, confocal

## Abstract

**Objectives:**

The objective of this study was to evaluate the bonding performance and the interfacial ultramorphology of an innovative self-curing restorative system compared with a conventional light-curing resin composite applied on dentin in etch-and-rinse (ER) or self-etch (SE) mode.

**Materials and Methods:**

Twenty cavities (class I) were prepared in sound dentin and restored using two materials: (1) CERAM ( *n* = 10; CERAM.X ONE, Dentsply Sirona) in combination with a universal adhesive system (PBU [Prime & Bond Universal]), or (2) STELA ( *n* = 10; Stela Automix, SDI) in combination with its adhesive primer. Half of the specimens from each group were bonded in ER or SE mode. Specimens underwent microtensile bond strength testing after 24 hours of storage in artificial saliva. Failure mode was determined using a stereomicroscope, and fractographic analysis was performed using scanning electron microscopy. The interfacial ultramorphology/nanoleakage of the resin–dentin slabs was analyzed through dye-assisted confocal microscopy.

**Statistical Analysis:**

For quantitative analysis, bond strength values (in MPa) were assessed for normality and variance using Kolmogorov–Smirnov and Levene's tests, followed by ANOVA based on restorative material and adhesive bonding protocol, with Fisher's least significant difference post hoc test (α = 5%).

**Results:**

SE groups exhibited significantly lower bond strength (17.4 MPa for CERAM; 26.2 MPa for STELA) compared with ER groups (35.8 MPa for CERAM; 33.6 MPa for STELA) ( *p* < 0.05). CERAM applied in SE mode showed significantly lower bond strength compared with STELA applied in SE mode. Furthermore, CERAM applied in SE mode was the only group presenting a pre-test failure rate (27%). The failure mode was predominantly mixed in ER groups and adhesive in SE groups. Nanoleakage was observed clearly in the CERAM groups applied in both ER and SE modes but was less evident in the STELA groups.

**Conclusion:**

The new self-curing material (STELA) used in SE or ER may represent a promising clinical option to provide adequate interfacial adaptation and strong bonding to dentin when restoring deep class I cavities. The use of conventional adhesives in deep class I cavities may generate resin-dentin interfaces characterized by gaps and leakages.

## Introduction

The field of adhesive dentistry has become an essential component of dental practice since Dr. Buonocore published his work elucidating the principles of acid etching of enamel. 1 Subsequently, with the decline in the use of dental amalgam and the shift toward minimally invasive operative dentistry, resin composites have emerged as the preferred choice for direct restorative interventions, 2 even in dentin, where adhesive challenges are more pronounced compared with enamel, mainly due to its inorganic composition and relatively high water content. 3 As a result, significant evolution in bonding agents and restorative materials has occurred. 4 Such improvements have led to a simplification in their clinical application protocols, 5 which is highlighted by a transition from etch-and-rinse (ER) multi-bottle/step systems to universal one-bottle adhesives. 4 5

Despite this progress, some challenges persist, such as polymerization shrinkage during light-curing procedures. 6 7 The integrity of the seal between resin composite materials and dental hard tissues, such as dentin and enamel, can be compromised due to the volumetric reduction of the restorative material during the polymerization step. 3 8 Recently, an innovative self-curing restorative system (STELA, SDI Ltd, Australia) has become available commercially. Unlike conventional light-cured materials, STELA is a self-curing resin-based bulk-fill material 9 that is used in combination with a proprietary adhesive primer, which does not require light-curing, but it undergoes polymerization upon contact with the restorative material. 10

To understand the real performance and how to utilize correctly this new type of new self-curing resin-based bulk-fill system in clinical practice, it is essential to assess at least the immediate microtensile bond strength (MTBS) and its interfacial adaptation when applied in ER and self-etch (SE) and compare it to conventional restorative materials.

Thus, this study aimed at comparing the immediate bonding performance and interfacial ultramorphology of STELA to a conventional light-cured resin composite (CERAM) used in combination with a modern universal adhesive system. All tested materials were applied according to the manufacturer's instructions and used in combination with their respective adhesive systems, which were applied in SE or ER mode. The null hypothesis was that there would be no significant difference in bonding performance and interfacial adaptation between the two tested resin composites when applied in SE or ER modes.

## Materials and Methods

### Sample Preparation

Twenty noncarious human third molars were collected and approved by the ethical committee of the host institution (ethical approval number: CEEI22/309, University CEU Cardenal Herrera, Valencia, Spain). The teeth were stored in distilled water at 4°C and used within 4 months of extraction. The crowns of each tooth were removed from their roots using a diamond-impregnated cutting disk (Isomet Diamond Wafering Blade, no. 11–4244, Buehler Ltd., Lake Bluff, United States) under continuous water cooling mounted on an automized sectioning machine working at 350 rpm (IsoMet 1000, Buehler Ltd., Lake Bluff, United States). Standard class I cavities were prepared in sound dentin (4 mm mesio-distal width × 3 mm buccolingual width × 4 mm deep), with margins located in the occlusal enamel and the cavity bottom ending in mid-coronal dentin. A high-speed air turbine handpiece with a diamond bur (882, Komet, Lemgo, Germany) was used to prepare the cavities; these were finished with fine diamond burs (8856, Komet, Lemgo, Germany), used under continuous water irrigation. The specimens were maintained in distilled water at 37°C (pH 6.7) for no longer than 1 hour before bonding and restorative procedures.

### Experimental Design and Restorative Procedures

Two groups ( *n* = 10 specimens/group) were created based on the restorative materials used in this study: (1) CERAM (CERAM.X ONE, Dentsply Sirona—Dentsply Caulk, Milford, Delaware, United States) was used as the control conventional light-cured resin composite in combination with the adhesive system (PBU [Prime & Bond Universal]); (2) STELA, a self-cure bulk-fill restorative system (Stela Automix, SDI Ltd, Bayswater, Australia), was used in combination with its proprietary adhesive system (Stela Primer, SDI Ltd.) that requires no photo-polymerization.

**Fig. 1:**
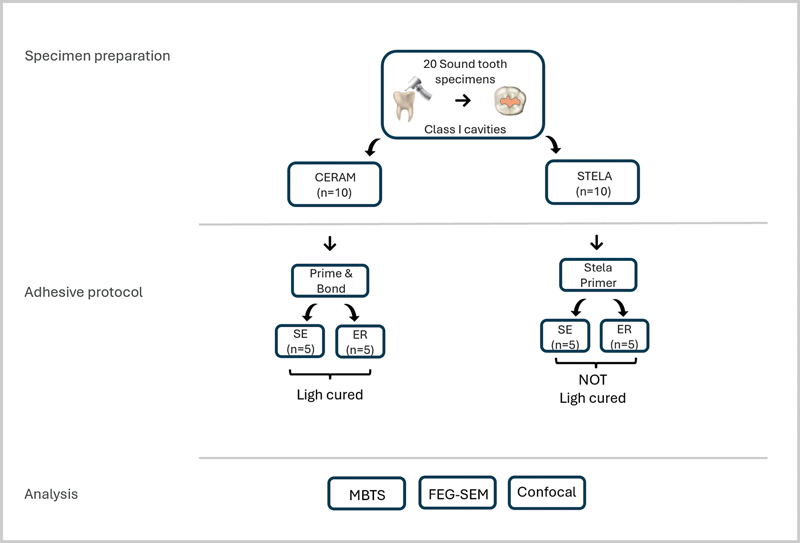
Schematic representation of the experimental design, demonstrating the allocation of specimens according to the methodological procedures. MTBS, microtensile bond strength; FIB-SEM, focused ion beam scanning electron microscope; Confocal microscopy.

As per the experimental design of this study ( Fig. 1 ), specimens were divided into two sub-groups ( *n* = 5 specimens/group) based on the bonding procedure (SE or ER mode). The adhesives applied in SE mode were brushed into the cavities using a microbrush for 20 seconds, followed by 5 seconds of air drying to evaporate the solvent. In the ER groups, a 37% orthophosphoric acid gel was left undisturbed in dentin for 15 seconds, subsequently rinsed with distilled water (15 seconds) and the dentine surface was finally air-blotted to leave a moist surface. All materials and adhesive systems were used according to the manufacturer's instructions and light-cured (when necessary), through a light-emitting diode curing unit (Radii Plus, SDI) with a mono-wavelength of 470 nm. The irradiance of this unit was 1,200 mW/cm ^2^ , which was checked using a laboratory-grade spectral radiometer.

The specimens were finally restored with the test restorative materials as previously mentioned. The conventional CERAM resin composite was applied by layering two horizontal increments of 2 mm, which were separately light-cured for 30 seconds. The self-curing bulk-fill restorative system (STELA) was placed in a single increment (5 mm) and allowed self-cure at room temperature and pressure for 4 minutes. Table 1 includes the composition of the materials and the manufacturer's instructions for use.

**Table 1:** Description of the composition, application mode, and manufacturer of dental materials used in the present study

Group	Composition	Application	Manufacturer
**CERAM** (CERAM.X ONE, Dentsply)	Poly-urethanemethacrylate, bis-EMA, TEGDMA glycol dimethacrylate (TEGDMA), camphorquinone (CQ)/butylated hydroxyl toluene (BHT), barium glass and ytterbium fluoride fillers (YbF3)	• Apply the composite over the dentin in horizontal increments (1–2 mm thick), three increments at all • Light cure each increment separately for 20 seconds	Dentsply Sirona—Dentsply Caulk, Milford, Delaware, United States
**PBU** (Prime & Bond Universal)	PENTA (dipentaerythritol pentacrylate phosphate), 10-MDP (10-methacryloyloxydecyl dihydrogen phosphate), Active Guard Technology crosslinker. CQ/tertiary amine. Isopropanol, water	• Apply the adhesive on the surface and rub it for 20 seconds • Gently air-dry the adhesive for approximately 5 seconds for the solvent to evaporate • Light cure for 10 seconds	Dentsply Sirona—Dentsply Caulk, Milford, Delaware, United States
**STELA** (SDI STELA Automix)	Catalyst: barium-glass, glass, ytterbium trifluoride (YbF3), silica, urethane dimethacrylate, initiators, stabilizers Base: strontium fluoroaluminosilicate glass, ytterbium trifluoride agglomerates (YbF3), silica, calcium aluminate (Al _2_ CaO), urethane dimethacrylate, initiators, stabilizers	• Extrude paste into the cavity in a single increment (up to 5 mm thick), being careful not to trap air under the restoration. Slightly overfill to ensure good contact with the primer at the margin. Wait 4 minutes (self-cure)	SDI Limited, Australia
**STELA Primer**	10-MDP, dimethacrylates, methyl ethyl ketone (MEK), water, initiators, stabilizers.	• Apply STELA Primer onto prepared cavity surfaces and leave on cavity for 5 seconds. • Gently air-dry until you see no movement of the primer (2–3 seconds). Note: Do not light-cure. STELA Primer cures upon contact with STELA restorative material.	SDI Limited, Australia

### Specimen Preparation for Microtensile Bond Strength and Fracture Analysis (FIB-SEM) of Resin Composite–Dentin Interfaces

The specimens were sectioned serially (IsoMet 1000, Buehler Ltd., United States) after 24 hours of storage in artificial saliva at 37°C to create resin–dentin sticks (18–21 each tooth) with a dimension of 0.9 mm ^2^ . The enamel was removed by sectioning the specimens into sticks, ensuring no enamel was present on the bonding surfaces. All specimens were subsequently inspected under a stereoscopic microscope (×40) at approximately ×30 magnification to confirm the absence of enamel, defects at the interface, bubbles, or irregularities in the proximity of the resin–dentin bond. The sticks from each group were submitted to MTBS testing, by fixing them to a jig using a cyanoacrylate extra-hard glue and then stressed to failure in an MTBS testing device (BISCO Corp., United States). The tensile force was applied at a crosshead speed of 1 mm/min until failure occurred at the bonding interface. The maximum tensile load at failure (N) was divided by the respective cross-sectional areas of each stick and bond strength values were converted to MPa.

Fractographic analysis was performed using a stereomicroscope to examine the failure mode of each specimen (adhesive, mixed, or cohesive). Representative fractured specimens from each group were selected, mounted on stubs, gold-coated (MED 010, Balzers, Liechtenstein) and submitted to fractographic analysis using an ultra-high resolution analytical focused ion beam scanning electron microscope (FIB-SEM, Thermo Scientific Scios 2 DualBeam, Waltham, Massachusetts, United States) in secondary electron mode.

### Confocal Microscopy Assessment of Resin Composite–Dentin Interface Morphology/Nanoleakage

Three resin composite–dentine slabs were selected from the center of each cavity specimen in each experimental group during the cutting procedures outlined in the microtensile specimen preparation section. These specimens were polished for 30 seconds using 1,200-grit SiC papers, followed by an ultrasonic bath in distilled water for 3 minutes. The slabs were coated with varnish, leaving a 1 mm gap between the dentin at the composite and subsequently immersed in a Rhodamine B water solution (0.15 wt.%, pH 7) for 24 hours. The specimens were then rinsed with distilled water and immersed in an ultrasonic bath for 3 minutes. Finally, the slabs were again submitted to polishing for 30 seconds on each side using 1,200-grit SiC papers, followed by a final ultrasonic bath for 5 minutes.

All specimens underwent confocal microscopy analysis using an Olympus FV1000 system (Olympus Corp., Tokyo, Japan) equipped with a 40 ×/1.4 NA oil-immersion lens and illuminated with a 543 nm LED. Reflection and fluorescence images were captured with a 1-µm z-step to optically section the specimens up to a depth of 20 µm below the surface. The z-axis scan of the interface surface was pseudo-colored arbitrarily for improved exposure and assembled into single-image projections (Fluoview Viewer, Olympus Corp., Tokyo, Japan). The system configuration remained consistent throughout the entire study. The bonded-dentin interface was examined, with images randomly obtained from three different zones of the bonding interface; micrographs with the most representative morphological features identified along the resin–dentin interfaces were collected.

### Statistical Analysis

A quantitative analysis of bond strength values in MPa was performed for both the evaluation of the normality distribution and variance homogeneity using Kolmogorov–Smirnov and Levene's tests. Analysis of variance (ANOVA) was accomplished by considering factors such as restorative material and adhesive bonding protocol. Finally, the data were processed using Fisher's LSD post-hoc test. The significance level was set at 0.05 and maintained throughout the entire analysis (Bioestat v.5.3; Instituto Mamirauá, Manaus, AM, Brazil).

## Results

### Microtensile Bond Strength and Fracture Analysis

The results of the MTBS test are presented in Table 2 . It was observed that after 24 hours of artificial saliva storage, significantly higher mean bond strength values were attained in the ER mode compared with the SE mode for both tested materials ( *p* < 0.05). However, CERAM exhibited the lowest bond strength in the SE mode (17.4 MPa), while STELA had a higher bond strength to dentin (26.2 MPa). Most of the specimens failed in adhesive and mixed modes during the test for both resin composites applied in SE mode. CERAM used in SE mode demonstrated a pre-test failure rate of 27% across all groups. Conversely, in the ER mode, STELA demonstrated comparable results to the conventional CERAM composite (33.6 and 35.8 MPa, respectively; *p* < 0.05), with mixed mode failure being predominant in both cases.

**Table 2:** The results show the mean (±SD) of the µTBS (MPa) to dentine and the percentage (%) of the failure mode analysis [adhesive/mixed/cohesive]

	SE 24H	ER 24H
**CERAM** (27%/0%)	17.4 ± 4.7 **A**	35.8 ± 2.4 **a** a
	[57/43/0]	[25/55/20]
**STELA** **(0%/0%)**	26.2 ± 2.9 **B**	33.6 ± 2.1 **a** a
	[59/41/0]	[36/60/4]

Note: The percentages (%) of pre-test failure values before µTBS for each tested group are also depicted (SE%/ER%). A similar uppercase letter indicates no significance (in columns) between the resin composites applied on dentine bonded in SE mode. A similar lowercase letter indicates no significance (in columns) between the resin composites applied on dentine bonded in ER mode.

a This symbol indicates a significant difference (in row) between the results of a specific material (CERAM or STELA) applied in SE or ER mode.

**Fig. 2:**
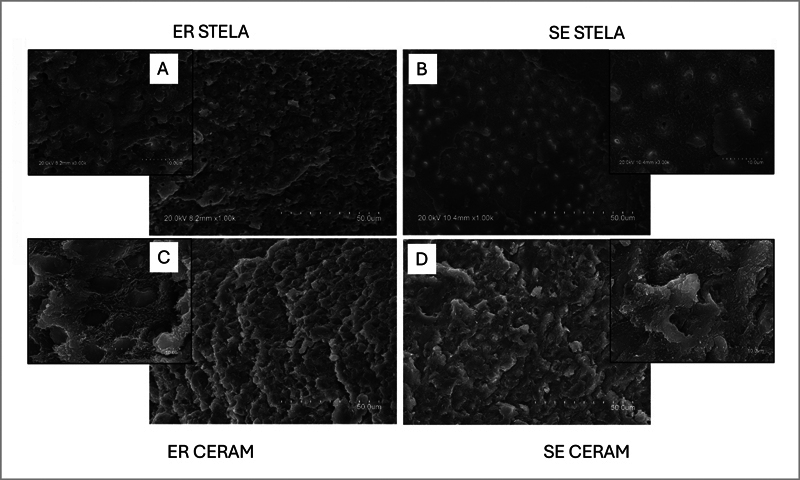
SEM fractographic analysis of the specimens tested at 24 hours. ( **A** ) STELA composite with STELA Primer in ER mode shows fractures beneath the hybrid layer, few exposed collagen fibrils, and occluded dentin tubules. ( **B** ) STELA in SE mode displays fractures within the bonding interface, no exposed collagen, and occluded dentin tubules. ( **C** ) CERAM composite with PBU in ER mode exhibits fractures within the hybrid layer, with exposed collagen fibrils and fractured resin tags. ( **D** ) CERAM with PBU applied in SE mode shows residual smear layer on dentin surface and inside tubules. ER, etch and rinse; PBU, Prime & Bond Universal; SE, self-etching; SEM, scanning electron microscopy.

The results of the fractographic analysis performed after MTBS testing of the tested materials are depicted in Fig. 2 . Specimens prepared with STELA composite using STELA Primer in ER mode, which mainly failed in mixed mode (60%), showed fractures beneath the hybrid layer, with few exposed acid-etched collagen fibrils and occasionally occluded dentin tubules ( Fig. 2A ). Specimens of STELA applied in SE mode exhibited fractures within the bonding interface, with no exposed collagen fibrils present and dentin tubules still occluded by the smear layer ( Fig. 2B ).

Specimens prepared with the CERAM composite and PBU adhesive in ER mode, which failed mainly in mixed mode (55%), predominantly exhibited fractures within the hybrid layer. These fractures revealed exposed acid-etched collagen fibrils and fractured resin tags inside dentin tubules ( Fig. 2C ). Conversely, specimens prepared with the same resin composite and PBU in SE mode, which failed mainly in adhesive mode (57%), often showed a residual smear layer on the dentin surface and within dentin tubules ( Fig. 2D ).

### Confocal Microscopy Assessment of Resin Composite–Dentin Interface Morphology

**Fig. 3:**
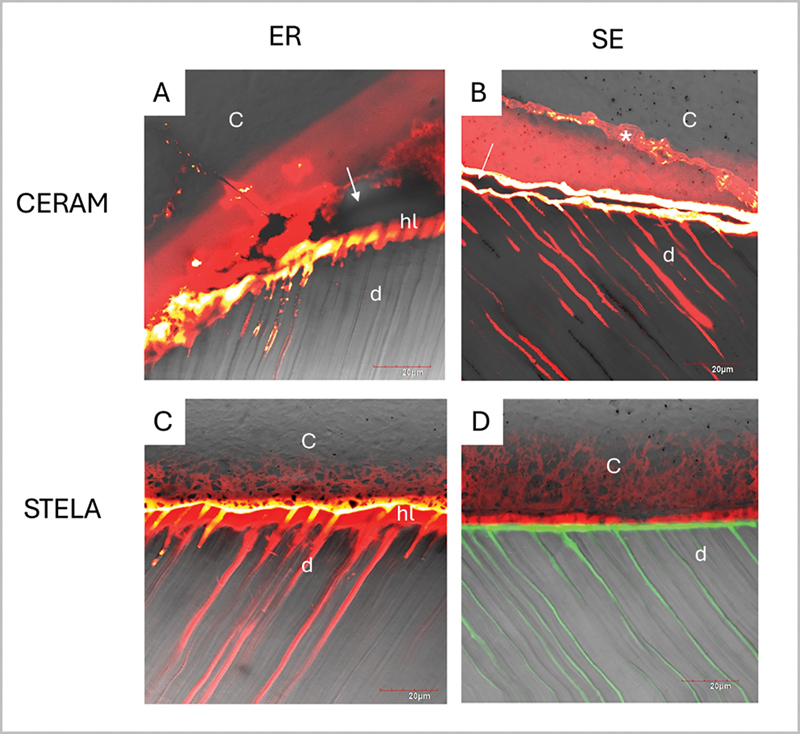
Confocal microscopy images of the resin–dentin interfaces tested at 24 hours. ( **A** ) Specimens with CERAM composite and PBU adhesive applied in ER mode exhibited large gaps and voids within the resin composite (white arrow), along with fluorescent dye infiltration in the hybrid layer. Some microcracks within the resin composite were also infiltrated by the dye (*), attributed to polymerization shrinkage. ( **B** ) Specimens with CERAM composite and PBU adhesive in SE mode often displayed gaps along the dentin–composite interface (white arrow), allowing fluorescent dye accumulation inside dentin tubules. ( **C** ) The resin–dentin interface formed by STELA Primer in ER mode showed no gaps, but positive dye infiltration. ( **D** ) The resin–dentin interface formed by STELA Primer in SE mode showed a compact layer between dentin and resin composite without gap formation. (c) Composite; (d) dentin; (hl) hybrid layer. ER, etch and rinse; PBU, Prime & Bond Universal; SE, self-etching.

The results of confocal microscopy for the tested materials are illustrated in Fig. 3 . The specimens created with CERAM composite applied in dentin, in combination with the PBU adhesive in ER mode, consistently exhibited gaps and voids within the resin composite ( Fig. 3A ). The specimens created with CERAM composite applied in dentin in combination with the PBU adhesive in SE mode often displayed gaps along the dentin–composite interface. Indeed, fluorescent dye nanoleakage in hybrid layer was observed, along with the presence of microcracks in the resin composite infiltrated by the dye. This was probably attributed to polymerization shrinkage of the CERAM and a lack of adhesion performance of the adhesive used in SE mode ( Fig. 3B ).

In the STELA specimens, gaps and voids were mainly absent ( Fig. 3C, D ). However, within the resin–dentin interface created by the application of STELA Primer in ER mode, fluorescent dye infiltration/nanoleakage was detected at the bonding layer ( Fig. 3C ). Additionally, the resin–dentin interface created by the application of STELA Primer in SE mode was characterized by a compact layer between the dentin and the material ( Fig. 3D ).

## Discussion

The comparative evaluation conducted in this *in vitro* study provides valuable insights into the bonding interface between conventional light-cured resin composite and a newly introduced self-curing resin-based restorative material. Considering the results, the null hypothesis that there would be no differences in immediate bond strength and interfacial adaptation between STELA and the conventional composites when applied in SE or ER mode was partially rejected. No significant differences were observed in bond strength values between the two materials applied in ER mode ( *p* > 0.05), indicating that both materials performed similarly under these conditions. However, bond strength values were consistently lower when using the SE mode for both materials, which aligns with existing literature highlighting the challenges of achieving optimal bond strength with SE strategies in high C-factor cavities. 11 12 13 These results are consistent with previous findings, 10 where STELA demonstrated superior bond strength in similar testing conditions, outperforming other conventional composites tested. In that study, STELA achieved bond strengths of 23.2 MPa in SE mode and 32.4 MPa in ER mode after 24 hours of storage, showing comparable performance to the conventional 3M-CTR composite (22.4 MPa in SE mode and 38.8 MPa in ER mode), and significantly outperforming the 3M-BULK composite in SE mode (9.9 MPa), which experienced a higher number of pre-failures.

In the present study, STELA showed significantly higher bond strength values (26.2 MPa) in SE mode compared with CERAM (17.4 MPa; *p* < 0.05). The data suggest that STELA's formulation may offer better bonding performance using an SE protocol compared with conventional universal adhesive and composites used in SE mode. This may be potentially correlated to its unique chemical composition 9 and polymerization mechanism, 14 which could enhance adhesive infiltration and interaction with the dentin substrate, thereby reducing shrinkage stress on the bond interface. 10 Moreover, the previous findings indicate that STELA exhibits lower susceptibility to bond strength reduction over time compared with other bulk-fill materials. 10

For interfacial adaptation, the ultramorphological/nanoleakage analysis revealed gaps and voids in the conventional resin composite ( Fig. 3A, B ), indicative of poor adaptation, as well as possible high shrinkage stress. In contrast, STELA provided superior adaptation to the dentin, forming a compact layer with no gaps and less evident nanoleakage ( Fig. 3C, D ). The basic composition of conventional dental resin comprises a combination of hydrophobic resin monomers, inorganic fillers, and photoinitiators. 15 These components initiate polymerization through a free-radical reaction upon exposure to visible light. In class I cavities, the influence of the C-factor on polymerization shrinkage dynamics is pronounced, resulting in volumetric reduction of the restorative material. 16 17 18 This leads to significant implications for marginal integrity, potentially fostering gap formation at the adhesive interface and exacerbating hydrolytic and enzymatic degradation, 19 20 21 thereby increasing the risk of development of caries associated with restorations and sealants (CARS). 22 23

A high C-factor class I cavity was chosen to simulate clinical conditions for resin composite use, testing the materials under challenging clinical protocols. 16 This factor may have influenced the bond strength to the extent that polymerization shrinkage caused gap formation. 2 It is important to note that *in vitro* studies may not fully replicate the complex conditions of the oral environment, such as thermal and mechanical stresses, substrate variability, and clinical technique sensitivity, which could limit the direct extrapolation of these findings to clinical practice. However, the alterations in polymerization dynamics presented in STELA composite may have attenuated the stress produced by shrinkage and established chemical adhesion to dentin. 19 24 Furthermore, STELA was the only material without pre-test failures when applied with both adhesive strategies ( Table 2 ), suggesting that this material could withstand polymerization stress.

STELA is a self-cure, resin-based, bulk-fill material that claims to be a potential amalgam replacement material but with improved aesthetic properties. 25 It represents a recent generation of resin composites that combine the restorative ability of a material with chemically adhesive potential. 2 During the chemical adhesion process, a prolonged pre-gel phase may decelerate polymerization thus reducing shrinkage stress build-up, 9 unlike photoactivated materials. In this regard, its chemical–physical characteristics may be aligned with glass-ionomer cement, which not only exhibits bio-interactive properties but has also been shown to attenuate stress generated by overlying resin composite shrinkage and maintain bonding performance, when used as a base material in a deep cavity. 26 27

Moreover, STELA may provide ions to the interface 10 due to its filler composition, which facilitates dynamic ionic exchange (e.g., strontium, silica, calcium aluminate). 28 29 Hence, apart from its “mineral deposition” properties that induce ion exchange, warranting further investigation, this study focused on *in vitro* testing of primary properties such as bonding performance and interfacial analysis, which remain essential. However, additional clinical studies are needed to validate these findings and assess the long-term clinical performance of these materials *in vivo* .

It is also important to discuss the fractographic analysis, with STELA ( Fig. 2A, B ) displaying few exposed collagen fibrils and occluded dentin tubules, possibly due to mineral deposition. In contrast, the CERAM composite exhibited exposed collagen fibrils that would be more prone to degradation. 8 When collagen fibrils are exposed and not completely infiltrated by adhesive monomers, 30 they become susceptible to hydrolytic and enzymatic degradation, 20 which compromises the integrity of the resin–dentin bond over time. 31 32 At clinical level, this degradation can lead to increased microleakage at the restoration margins, allowing the ingress of oral fluids, bacteria, and other contaminants. 3 Consequently, patients may experience postoperative sensitivity, and increased microleakage can significantly elevate the risk of CARS, as bacteria can infiltrate the gaps between the tooth and restoration. 23

Universal adhesive systems have been developed to simplify and address such issues, including clinical technique sensitivity of clinical application protocols. These systems can be applied in both SE and ER modes. One of the key components of universal adhesive systems is the monomer 10-MDP (10-methacryloyloxydecyl dihydrogen phosphate). The presence of 10-MDP is crucial because it forms a strong chemical bond with calcium ions in hydroxyapatite, 33 resulting in the formation of a stable hybrid layer, as illustrated in Fig. 3 .

Considering the application mode of adhesives, they correlated with the failure modes observed at the bond interface. In the SE groups, adhesive and mixed failure modes predominate, indicating weaker cohesive strength within the interface. Conversely, the ER groups exhibited predominantly mixed failure modes, suggesting superior interfacial integrity and cohesive strength between the resin composite and dentin. This highlighted the critical role of adhesive protocol selection in optimizing bonding performance, where the choice of restorative material and bonding protocol significantly influences the longevity and clinical success of restorations. 12 34 35 In this study, the ER mode demonstrated superior performance. However, it is important to consider that resin-dentin interfaces created using simplified adhesive systems applied in ER mode are more prone to degradation over time compared with those created with the same systems applied in SE mode. 12 34 35 Indeed, the deterioration of collagen fibrils within the hybrid layer plays a critical role in reducing the bonding effectiveness of adhesive systems, particularly when used on acid-etched dentin. Research has shown that phosphoric acid in dental etchants and acidic functional monomers represent the key in removing minerals from collagen, which in turn activates proteolytic enzymes such as matrix metalloproteinases and cysteine cathepsins. 35 36 These enzymes, especially during aging, can degrade dentin collagen fibrils that are not protected by resin, starting at the base of the hybrid layer. This degradation leads to the formation of gaps at the resin–dentin interface, increasing microleakage and the likelihood of recurrent caries. 37

The high organic and water content in dentin makes this substrate a less ideal for resin-bonding systems compared with enamel. Additionally, hydrolytic degradation of the polymeric matrix at the resin-dentin interface, especially with simplified adhesives in the ER mode, can occur due to water absorption and incomplete solvent evaporation, particularly under simulated pulpal pressure. 38 39 40 Significant water absorption can also cause mild acidic adhesives to release protons, which may accelerate the degradation of the resin-dentin interface. 40 41 42

The first limitation of this study is that it was conducted *in vitro* , which means it may not fully replicate the complex conditions of the oral environment, such as thermal and mechanical stresses, substrate variability, and clinical technique sensitivity. This limits the direct extrapolation of the findings to clinical practice. Moreover, this study focused on high C-factor class I cavities, which may not represent all clinical scenarios. The influence of polymerization shrinkage and gap formation might differ in other cavity configurations. It will be necessary to perform long-term performance studies on the resistance to hydrolytic and enzymatic degradation also in alternative cavity configurations.

Therefore, our future directions are focused on conducting long-term clinical studies to validate the *in vitro* findings and assess the real-world performance of STELA and other resin-based restorative materials. Moreover, we will investigate the bonding performance and interfacial adaptation of the materials in various cavity configurations to better understand their behavior in different clinical scenarios and perform aging studies to evaluate the durability of the bond over time, including resistance to hydrolytic and enzymatic degradation. It will be also necessary to explore the underlying mechanisms of STELA's superior bonding performance, particularly its chemical composition and polymerization dynamics, to optimize its formulation and application protocols. Finally, it is important to assess patient-centered outcomes, such as postoperative sensitivity and the incidence of CARS, to ensure the clinical success and patient satisfaction with STELA.

## Conclusion

This study provided *in vitro* evidence on the newly introduced self-curing restorative system (STELA), which demonstrated some superior bonding capabilities, particularly in SE protocols when compared with conventional light-cured resin composites. Thanks to its distinct chemical formulation and polymerization mechanism, STELA may achieve a proper adhesion to dentin with reduced risk of pre-failure, as well as gaps and voids at the interface. This makes STELA a viable alternative to conventional light-cured resin composites for the restoration of a high C-factor restorative cavities.

## References

[1] Buonocore M G A simple method of increasing the adhesion of acrylic filling materials to enamel surfaces J Dent Res 1955 34 06 849 853 13271655 10.1177/00220345550340060801

[2] Inglês M Vasconcelos E Cruz J Mano Azul A Polido M Delgado A HS Comparative assessment of different pre-treatment bonding strategies to improve the adhesion of self-adhesive composites to dentin Polymers (Basel) 2022 14 19 3945 36235894 10.3390/polym14193945 PMC9570807

[3] Perdigão J Current perspectives on dental adhesion: (1) dentin adhesion - not there yet Jpn Dent Sci Rev 2020 56 01 190 207 34188727 10.1016/j.jdsr.2020.08.004 PMC8216299

[4] Cadenaro M Josic U Maravić T Progress in dental adhesive materials J Dent Res 2023 102 03 254 262 36694473 10.1177/00220345221145673

[5] Sofan E Sofan A Palaia G Tenore G Romeo U Migliau G Classification review of dental adhesive systems: from the IV generation to the universal type Ann Stomatol (Roma) 2017 8 01 1 17 28736601 10.11138/ads/2017.8.1.001 PMC5507161

[6] Van Ende A De Munck J Lise D P Van Meerbeek B Bulk-fill composites: a review of the current literature J Adhes Dent 2017 19 02 95 109 28443833 10.3290/j.jad.a38141

[7] Ghavami-Lahiji M Hooshmand T Analytical methods for the measurement of polymerization kinetics and stresses of dental resin-based composites: a review Dent Res J (Isfahan) 2017 14 04 225 240 28928776 10.4103/1735-3327.211628 PMC5553250

[8] Betancourt D E Baldion P A Castellanos J E Resin-dentin bonding interface: mechanisms of degradation and strategies for stabilization of the hybrid layer Int J Biomater 2019 2019 5.268342E6 10.1155/2019/5268342 PMC6378048 30853990

[9] Thadathil Varghese J Raju R Farrar P Prentice L Prusty B G Comparative analysis of self-cure and dual cure-dental composites on their physico-mechanical behaviour Aust Dent J 2024 69 02 124 138 38131257 10.1111/adj.13004

[10] Pires P M de Almeida Neves A Lukomska-Szymanska M Farrar P Cascales ÁF Sauro S Bonding performance and interfacial adaptation of modern bulk-fill restorative composites after aging in artificial saliva: an in vitro study Clin Oral Investig 2024 28 02 132 10.1007/s00784-024-05525-5 38308668

[11] Cardoso G C Nakanishi L Isolan C P Jardim P DS Moraes R R Bond stability of universal adhesives applied to dentin using etch-and-rinse or self-etch strategies Braz Dent J 2019 30 05 467 475 31596331 10.1590/0103-6440201902578

[12] Fan-Chiang Y S Chou P C Hsiao Y W Optimizing dental bond strength: insights from comprehensive literature review and future implications for clinical practice Biomedicines 2023 11 11 2995 38001996 10.3390/biomedicines11112995 PMC10669570

[13] Sauro S Pashley D H Strategies to stabilise dentine-bonded interfaces through remineralising operative approaches: state of the art Int J Adhes Adhes 2016 69 39 57

[14] Van Meerbeek B Frankenberger R Editorial: on our way towards self-adhesive restorative materials? J Adhes Dent 2019 21 04 295 296 31432043 10.3290/j.jad.a43044

[15] Pratap B Gupta R K Bhardwaj B Nag M Resin based restorative dental materials: characteristics and future perspectives Jpn Dent Sci Rev 2019 55 01 126 138 31687052 10.1016/j.jdsr.2019.09.004 PMC6819877

[16] Eichler E Vach K Schlueter N Jacker-Guhr S Luehrs A K Dentin adhesion of bulk-fill composites and universal adhesives in class I-cavities with high C-factor J Dent 2024 142 104852 38244909 10.1016/j.jdent.2024.104852

[17] Ghulman M A Effect of cavity configuration (C factor) on the marginal adaptation of low-shrinking composite: a comparative ex vivo study Int J Dent 2011 2011 159749 21949664 10.1155/2011/159749 PMC3178442

[18] Wang Z Chiang M Y Correlation between polymerization shrinkage stress and C-factor depends upon cavity compliance Dent Mater 2016 32 03 343 352 26778403 10.1016/j.dental.2015.11.003

[19] Burrer P Par M Fürer L Effect of polymerization mode on shrinkage kinetics and degree of conversion of dual-curing bulk-fill resin composites Clin Oral Investig 2023 27 06 3169 3180 10.1007/s00784-023-04928-0 PMC10264464 36869923

[20] Mazzoni A Scaffa P Carrilho M Effects of etch-and-rinse and self-etch adhesives on dentin MMP-2 and MMP-9 J Dent Res 2013 92 01 82 86 23128110 10.1177/0022034512467034 PMC3521453

[21] Mokeem L S Garcia I M Melo M A Degradation and failure phenomena at the dentin bonding interface Biomedicines 2023 11 05 1256 37238927 10.3390/biomedicines11051256 PMC10215576

[22] Bourbia M Ma D Cvitkovitch D G Santerre J P Finer Y Cariogenic bacteria degrade dental resin composites and adhesives J Dent Res 2013 92 11 989 994 24026951 10.1177/0022034513504436 PMC3797536

[23] Kermanshahi S Santerre J P Cvitkovitch D G Finer Y Biodegradation of resin-dentin interfaces increases bacterial microleakage J Dent Res 2010 89 09 996 1001 20505047 10.1177/0022034510372885 PMC3318074

[24] Fehrenbach J Isolan C P Münchow E A Is the presence of 10-MDP associated to higher bonding performance for self-etching adhesive systems? A meta-analysis of in vitro studies Dent Mater 2021 37 10 1463 1485 34456050 10.1016/j.dental.2021.08.014

[25] Yao C Ahmed M H Zhang F Structural/chemical characterization and bond strength of a new self-adhesive bulk-fill restorative J Adhes Dent 2020 22 01 85 97 32030379 10.3290/j.jad.a44000

[26] Boroujeni P M Mousavinasab S M Hasanli E Effect of configuration factor on gap formation in hybrid composite resin, low-shrinkage composite resin and resin-modified glass ionomer J Investig Clin Dent 2015 6 02 156 160 10.1111/jicd.12082 24415719

[27] Naoum S J Mutzelburg P R Shumack T G Thode D Martin F E Ellakwa A E Reducing composite restoration polymerization shrinkage stress through resin modified glass-ionomer based adhesives Aust Dent J 2015 60 04 490 496 25476699 10.1111/adj.12265

[28] Moshaverinia M Navas A Jahedmanesh N Shah K C Moshaverinia A Ansari S Comparative evaluation of the physical properties of a reinforced glass ionomer dental restorative material J Prosthet Dent 2019 122 02 154 159 31326149 10.1016/j.prosdent.2019.03.012

[29] Pires P M Neves A A Makeeva I M Contemporary restorative ion-releasing materials: current status, interfacial properties and operative approaches Br Dent J 2020 229 07 450 458 33037365 10.1038/s41415-020-2169-3

[30] Liu Y Tjäderhane L Breschi L Limitations in bonding to dentin and experimental strategies to prevent bond degradation J Dent Res 2011 90 08 953 968 21220360 10.1177/0022034510391799 PMC3148178

[31] Frassetto A Breschi L Turco G Mechanisms of degradation of the hybrid layer in adhesive dentistry and therapeutic agents to improve bond durability–a literature review Dent Mater 2016 32 02 e41 e53 26743967 10.1016/j.dental.2015.11.007

[32] Tjäderhane L Dentin bonding: can we make it last? Oper Dent 2015 40 01 4 18 25615637 10.2341/14-095-BL

[33] Carrilho E Cardoso M Marques Ferreira M Marto C M Paula A Coelho A S 10-MDP based dental adhesives: adhesive interface characterization and adhesive stability-a systematic review Materials (Basel) 2019 12 05 790 30866488 10.3390/ma12050790 PMC6427605

[34] Carvalho R M Manso A P Geraldeli S Tay F R Pashley D H Durability of bonds and clinical success of adhesive restorations Dent Mater 2012 28 01 72 86 22192252 10.1016/j.dental.2011.09.011 PMC3863938

[35] Amin F Fareed M A Zafar M S Khurshid Z Palma P J Kumar N Degradation and stabilization of resin-dentine interfaces in polymeric dental adhesives: an updated review Coatings 2022 12 1094

[36] Sebold M Giannini M André C B Bonding interface and dentin enzymatic activity of two universal adhesives applied following different etching approaches Dent Mater 2022 38 06 907 923 35289283 10.1016/j.dental.2022.03.001

[37] Josic U D'Alessandro C Miletic V Clinical longevity of direct and indirect posterior resin composite restorations: an updated systematic review and meta-analysis Dent Mater 2023 39 12 1085 1094 37827872 10.1016/j.dental.2023.10.009

[38] Yoshihara K Hayakawa S Nagaoka N Okihara T Yoshida Y Van Meerbeek B Etching efficacy of self-etching functional monomers J Dent Res 2018 97 09 1010 1016 29554434 10.1177/0022034518763606

[39] Cuevas-Suárez C E da Rosa W LO Lund R G da Silva A F Piva E Bonding performance of universal adhesives: an updated systematic review and meta-analysis J Adhes Dent 2019 21 01 7 26 30799468 10.3290/j.jad.a41975

[40] Bacelar-Sá R Sauro S Abuna G Adhesion evaluation of dentin sealing, micropermeability, and bond strength of current hema-free adhesives to dentin J Adhes Dent 2017 19 04 357 364 28849795 10.3290/j.jad.a38866

[41] Feitosa V P Sauro S Zenobi W Degradation of adhesive-dentin interfaces created using different bonding strategies after five-year simulated pulpal pressure J Adhes Dent 2019 21 03 199 207 31093617 10.3290/j.jad.a42510

[42] Van Landuyt K L De Munck J Mine A Cardoso M V Peumans M Van Meerbeek B Filler debonding & subhybrid-layer failures in self-etch adhesives J Dent Res 2010 89 10 1045 1050 20631093 10.1177/0022034510375285

